# Resistance of Ion Exchange Membranes in Aqueous Mixtures of Monovalent and Divalent Ions and the Effect on Reverse Electrodialysis

**DOI:** 10.3390/membranes13030322

**Published:** 2023-03-10

**Authors:** Joost Veerman, Lucía Gómez-Coma, Alfredo Ortiz, Inmaculada Ortiz

**Affiliations:** 1 REDstack BV, Graaf Adolfstraat 35-G, 8606 BT Sneek, The Netherlands; 2 Departmento de Ingenierías Químicas y Biomolecular, Universidad de Cantabria, Av. Los Castros 46, 39005 Santander, Spain

**Keywords:** reverse electrodialysis, membrane conductivity, ion mobility, salinity gradient energy

## Abstract

Salinity gradient energy has gained attention in recent years as a renewable energy source, especially employing reverse electrodialysis technology (RED), which is based on the role of ion exchange membranes. In this context, many efforts have been developed by researchers from all over the world to advance the knowledge of this green source of energy. However, the influence of divalent ions on the performance of the technology has not been deeply studied. Basically, divalent ions are responsible for an increased membrane resistance and, therefore, for a decrease in voltage. This work focuses on the estimation of the resistance of the RED membrane working with water flows containing divalent ions, both theoretically by combining the one-thread model with the Donnan exclusion theory for the gel phase, as well as the experimental evaluation with Fumatech membranes FAS-50, FKS-50, FAS-PET-75, and FKS-PET-75. Furthermore, simulated results have been compared to data recently reported with different membranes. Besides, the influence of membrane resistance on the overall performance of reverse electrodialysis technology is evaluated to understand the impact of divalent ions in energy generation. Results reflect a minor effect of sulfate on the gross power in comparison to the effect of calcium and magnesium ions. Thus, this work takes a step forward in the knowledge of reverse electrodialysis technology and the extraction of salinity gradient energy by advancing the influence of divalent ions on energy recovery.

## 1. Introduction

**Figure 1:**
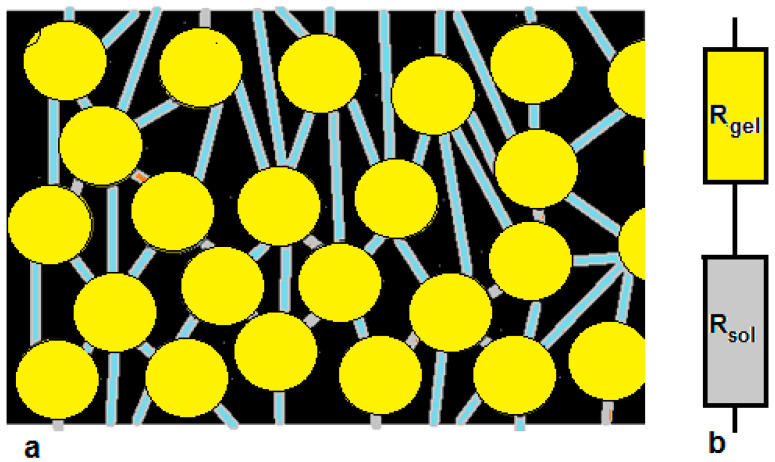
(**a**) Homogeneous ion exchange membrane. The small vesicles are filled with gel phase (yellow) containing fixed groups, water, and counter-ions, and a small amount of co-ions. The vesicles are interconnected by pores filled with water and dissolved ions (blue). (**b**) The one-thread model consisting of two resistances: R_gel_ with a constant resistance and R_sol_ with a concentration-dependent resistance [8].

Ion exchange membranes (IEMs) are of major importance in a huge variety of technologies, with their most important properties being permselectivity and resistance. These membranes are divided into heterogeneous membranes, which are made by mixing an ion exchange resin and a thermoplastic polymer, and homogeneous membranes, which have the ion exchange groups chemically bonded to a cross-linked backbone. Both heterogeneous and homogeneous membranes are not homogeneous at the micro-level: the charged groups form small vesicles of gel with water, and in the other places, it is the organic chains that stick together. These two phases are interspersed by an aqueous phase whose composition is reasonably believed to reflect the composition of the bulk in which the membrane is contained (Figure 1). 

Initially, it was assumed that homogeneous membranes were also homogeneous at the micro-level. The membrane properties could then be reasonably predicted with the theory of the Donnan equilibrium. However, it was Gierke et al. [1] who conducted in-depth studies on perfluorinated Nafion membranes. Using X-ray studies, these authors proposed a model consisting of adjacent vesicles covered on the inside with a gel layer and an aqueous solution in the center. In 2001, Kreuer [2] published an article comparing Nafion membranes with sulfonated polyketone-based CEMs and concluded that the structure of both types was broadly similar.

In 1993, Zabolotsky and Nikonenko introduced a micro-heterogeneous model to describe the properties of these membranes, in which they concluded that ion transport through the membrane alternately takes place via the gel phase and the solution phase [3]. With this model, Berezina et al. were able to model and measure the electrotransport of water [4]. Extensive measurements were then performed by Sarapulova et al. on a variety of homogeneous as well as heterogeneous membranes involving divalent ions [5,6,7]. A wide range of measurement methods were used for this characterization.

Applications that use IEMs, cation, or anion exchange membranes (CEMs and AEMs, respectively) are strongly affected by the properties of membranes, particularly counter/co-permselectivity and resistance. Especially for energy harvesting from different salinity gradients with reverse electrodialysis (RED) technology, membrane resistance is a key factor [9]. If an IEM is immersed in water, the fixed groups are counterbalanced by oppositely charged ions with the same concentration. These counter-ions are more or less trapped by the fixed groups. In this sense, Imai and Onishi introduced the term counter-ion condensation for this process [10]. Fixed groups and counter-ions are surrounded by water and form the gel phase of the membrane. If the IEM is in equilibrium with a NaCl solution, a small amount of co-ions (ions with the same charge sign as the fixed ions) penetrate into the gel phase. Since electroneutrality is required, these ions are also accompanied be an equal number of counter-ions. Thus, conduction in the gel phase is facilitated by the transport of: (i) condensed counter-ions, (ii) excess (or ‘free’) counter-ions, and (iii) co-ions. Kamcev et al. calculated, for the diffusion constant of condensed counter-ions, a value of about 2–2.5 times higher than for the excess counter-ions [11]. Assuming that the theory about the condensed ions is not purely hypothetical and that there is a real difference between the two types of counter-ions, it is still difficult to distinguish between them in practice. Moreover, the concentration of the excess ions is much lower than that of the condensed ions, so that their possible influence on the conductivity is marginal.

When treating natural water sources, Mg^2+^, Ca^2+^, and SO_4_^2−^, among other ions, may be present. In this context, previous works focused on the effect of divalent ions on the stack resistance and stack power density [12,13,14,15,16,17,18,19,20,21]. However, from measurements of stack resistance, it is difficult to discriminate the contribution of each of the four basic components of the stack, i.e., a CEM, an AEM, and the two feed water compartments, to the overall resistance. In contrast to this, the number of papers that focus on the effect of divalent ions on individual membranes is still very limited [6,7,15,18,22].

Nevertheless, to understand the principles of ion transport in an IEM, it is necessary to determine the molecular structure of such a membrane. It is striking that much research has been carried out on cation exchange resins and cation exchange membranes while significantly less work has been performed on anion exchange systems. Many efforts in the field have been performed to explain the structure of exchangers [23,24,25,26,27]. In this sense, these works performed ab initio calculations of systems of various monovalent and divalent cations together with fixed anions and determined the spatial structure of fragments around these ions. Soldatov et al. [27] reported calculations of the interactions between the counter-ions Na^+^ and K^+^ and the fixed groups of the carboxylate group (~COO^−^) and the sulfonate group (~SO_3_^−^).

**Figure 2:**
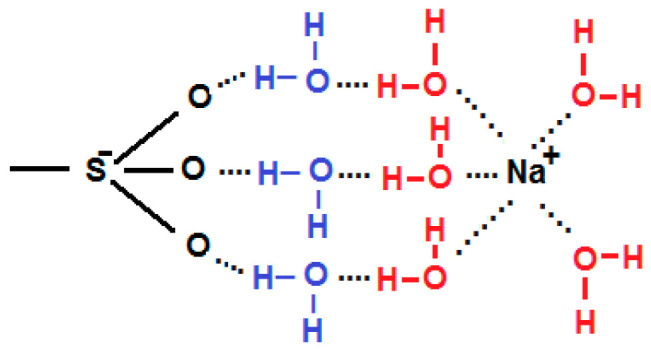
Interaction of the sodium and the sulfonate group, from Shaposhnik and Butyrkaya [24].

The nonexistence of a direct bond between a fixed sulfonate group and a sodium ion due to the strong hydration of the ion described by Soldatov et al. [27] agrees with the ab initio calculations of Shaposhnik and E. V. Butyrskaya [28]. These researchers found structures in which the sodium ion is connected to the sulfonate group through two intermediate water molecules. Both the sulfonate ion and the sodium ion are large enough to accommodate three adjacent bonds of this type, as seen in Figure 2.

Soldatov et al. [29] also performed ab initio calculations and arrived at roughly similar structures as Badessa and Shaposhnik [27]. In all these cases, the counter-ions are directly or indirectly bonded to the fixed groups. In the case of the trivalent ions, the bond distance is very short due to the large electrostatic action and the system can be compared with that in ion pairs.

In another publication, Badessa et al. [30] elaborated in more detail on the effect of the ion charge on the ion transport. The exchanging groups of conventional CEMs are in most cases singly charged. A monovalent counter-ion such as Na+ only needs to jump from one energy pit to another; in contrast, a divalent ion cannot jump in this way because it is forced to remain connected to one of the fixed groups throughout the transition.

The contribution to the conduction of each type of ion is the product of its concentration and mobility in the gel phase [3,4,31]. Mobilities of ions in aqueous solutions are well-known, but there is much uncertainty about the mobility in membranes. For lack of better results, some authors estimate these by a factor of 10 lower than in water [32].

In almost all published resistance measurements, the concentrations of the bulk solutions on each side of the membrane are equal, and only a few papers describe experiments with different concentrations [33,34]. Thus, in this work, we assume equal concentrations on both sides. Furthermore, most investigated divalent ions are Mg^2+^ and SO_4_^2−^; unfortunately, less research has been carried out on the influence of Ca^2+^. It appeared that Mg^2+^ has more negative influence on the power density in a RED stack than SO_4_^2−^ [18]. This effect is probably attributed to differences in: (i) the electrical interaction between the ion and the fixed groups, (ii) the steric hindrance of the ion in the membrane matrix (iii), the transport mechanisms of the ion, or (iv) the hydrophobicity of the ion. Another reason could be assumed due to the properties of the fixed groups. In functionalized membranes with ~SO_3_^−^ ions, most of the charge is located in the outer layer of the oxygen atoms, while in the case of functionalized membranes with ~NR_3_^+^, most of the charge is situated on the central nitrogen atom. This has consequences for coulombic interactions (ion–ion and ion-induced dipole).

In this context, this work first aims at advancing the knowledge of the contribution of divalent ions to membrane resistance, proposing different correlations as a function of the membrane nature, and second, at the quantification of the impact in salinity energy extraction, setting the basis for the future scale-up of the technology. It is important to mention that in this paper, we limit ourselves to resistance measurements regarding the individual membranes and power measurements in the complete RED stack. The ultimate goal is to gain insight into the entire behavior of the membrane under various conditions by means of a limited number of resistance measurements.

## 2. Materials and Methods

Model waters of different compositions were employed to prepare high- and low-concentration solutions, HCS and LCS, respectively [35,36]. For this purpose, sodium chloride (NaCl, assay > 99.5%) was acquired from Fisher Chemicals, magnesium chloride and calcium chloride were provided by Panreac (MgCl_2_·6H_2_O, assay > 98%), and sodium sulfate (Na_2_SO_4_, assay > 99%) was delivered by Scharlau. The divalent ions included were the typical ones presented in seawater (HCS) and wastewater treatment effluents (LCS).

In this work, 75 µm-thick and multivalent permeable membranes were employed (supplied by Fumatech, Bietigheim-Bissingen, Germany), which are exceptional to avoid fouling phenomena. Specifically, anion exchange membranes were based on bromide as the counter-ion and reinforced with polyethylene terephthalate, PET (FAS-75), and cation exchange membranes were developed with H^+^ as the counter-ion and were also supported with PET (FKS-75). All the tests were carried out, at least in triplicate, at 297 ± 1 K at 1 cm·s^−1^ as linear velocity. The experimental setup was described in detail in recent works [37,38].

The membrane resistance, one of the most critical parameters when divalent ions are present in water streams, was carefully determined through electrochemical impedance measurements (EIS). These trials were performed with the electrochemical workstation Zennium (Zahner, Kansas City, MO, USA) in potentiostatic mode. The system has six connectors: a counter electrode, a working electrode, a reference electrode, a working electrode sense point, and two probes. Before electrochemical measurements, the membranes were equilibrated in salt solutions for at least 24 h at 297 ± 1 K. EIS experiments were carried out following a direct-contact method, using an electrode surface area of 1.306 cm^2^ and generating an electric current in a frequency range of 100 Hz to 4 MHz, with a signal amplitude of 10 mV. For each solution, the experiment was replicated at least three times, changing the membrane sample. After each experiment, the electrode surface was properly cleaned with deionized water to remove the salt remnants.

Besides, the membranes were tested in a RED module (provided by Fumatech), assembled with 20 cell pairs (FAS-75 and FKS-75), using woven spacers of 270 µm with a porosity of 92.5% to demonstrate the membrane impact into salinity gradient energy extraction. The experimental setup used for this purpose has been described in detail in previous works [22,36,37,38].

In addition to the above experiments, other data have been collected from relevant publications dedicated to testing the resistance model (as described in Section 3) and have been used to validate the model. These data (concentrations and area resistances) are included in the Supplementary Materials.

## 3. Model of Ion Transport in Ion Exchange Membranes

The model developed in this paper describes the membrane resistance in terms of ionic concentrations and mobilities in the gel phase. A resistance measurement of a membrane with area *A* (cm^2^) and thickness d (cm) resulted in a resistance value R, measured in ohm (Ω). From these values, the specific resistance (R_spec_) and the area resistance (R_area_) can be estimated as:

(1) $$R_{spec}=R\frac{A}{d}\; [\Omega \cdot m]\;;\;R_{area}=R\cdot A\; [\Omega \cdot m^{2}]$$

The specific resistance, R_spec_, is a material property, independent of the membrane thickness, whereas the area resistance, R_area_, is a measure for the membrane performance. In membrane literature, R_area_ is usually expressed in Ω∙cm^2^.

To quantify the effect of the salt mixtures (with n different ions) on the membrane resistance, measurements for any given membrane should be performed at least with an n + 1 different bulk composition, and preferably much more. For simplicity, we expressed all concentrations in equivalents per liter (eq/L).

With the model, the relative mobilities of ions in a given membrane can be determined. The procedure presented here for a specific dataset involves the following steps:

i. Calculation of the concentrations of the ions in the gel from the known bulk concentrations using the Donnan theory.
ii. Assignment of known mobilities to the ions in the solution phase and provisional values to the ions in the gel phase.
iii. Assignment of provisional factors (‘phase factors’) to the conductivity of the gel part and the solution part.
iv. Estimation of the membrane resistance using the one-thread model [8].
v. Fitting the calculated resistances to the experimental values by adjusting the gel phase mobilities and the phase factors.

Assuming that the composition of the internal solution phase is the same as that of the bulk solution, the ion concentrations in the gel phase are obtained from the Donnan equilibrium:

(2) $$\frac{C_{i}^{gel}}{C_{i}^{sol}}={ [K_{i}]}^{z_{i}}$$

where Ci^sol^ corresponds to the different ion concentrations in the solution phase (and equal to the concentrations in the bulk solution) and Ci^gel^ corresponds to the concentration of these ions in the gel phase. K is the Donnan equilibrium constant and the exponent zi is the charge of ion i. For example, with a mixture of NaCl and MgCl_2_, there are three such relations with four unknowns: three ion concentrations and the value of K. To be able to solve this system, a fourth relation is needed, and for this purpose, the electroneutrality equation is used:

(3) $$CD+\underset{i}{\sum }C_{i}^{gel}z_{i}=0$$

with CD being the charge density of the membrane (in eq/L) and z_i_ the charge of species i (in this case, +1 or −1 because C is expressed in eq/L). The charge density can be achieved from the published membrane properties: swelling degree (SD), the ion exchange capacity (IEC), and the density of the dry membrane (ρ):

(4) $$CD=\rho \frac{IEC}{SD}$$

For practical use, ρ = 1 kg/L can used. According to Figure 1b, the total membrane resistance (R_mem_) is the sum of the gel contribution (R_gel_) and the solution contribution (R_sol_):

(5) $$R_{mem}=R_{gel}+R_{sol}$$

The resistance can be expressed in Ω (resistance), Ω∙m (specific resistance), or Ω∙m^2^ (area resistance). The conductivity, κ (S/m), of an ionic solution can be calculated as:

(6) $$\kappa =F\underset{i}{\sum }C_{i}\lambda_{i}$$

where F is the Faraday constant (96,485 C/mol), C_i_ is the concentration of the ionic particle i (eq/m^3^), and λ _i_ is its ionic mobility (m^2^∙s^−1^∙V^−1^). Ionic mobilities in aqueous solution are well-known and summarized in Table 1.

It should be noted that the mobility, λ_i_, and the diffusion constant, D_i_, of ions are closely related [40]:

(7) $$D_{i}=\frac{kT}{Q_{i}}\lambda_{i}$$

where k (J∙K^−1^) stands for the Boltzmann constant, Q (C) for the charge, and T (K) for the temperature. For a sodium ion at 298 K, this expression yields D = 1.33∙10^−9^ m^2^/s. The resistance, R_sol_ (Ω), of the solution part of a membrane is then:

(8) $$R_{sol}=\frac{Rf_{sol}}{\sum_{i}C_{i}^{sol}\mu_{i}^{sol}}$$

The values of the fitting factors Rf_sol_ and R_fgel_ depend on the structural characteristics of the membrane (fraction of solution and gel phases, tortuosity, membrane thickness, etc.), and C_i_^sol^ stands for the concentration of ion i in the gel phase. A similar expression can be derived for the gel part of the resistance, resulting in the following expression for the total membrane resistance, R_mem_:

(9) $$R_{mem}=\frac{Rf_{gel}}{\sum_{i}C_{i}^{gel}\mu_{i}^{gel}}\;+\frac{Rf_{sol}}{\sum_{i}C_{i}^{sol}\mu_{i}^{sol}}$$

Following this procedure, it is not possible to determine the individual value of Rf_gel_ together with the ion mobilities in the gel phase of the membrane. However, the relative value of these mobilities, φ_i_^gel^, can be calculated by setting Rf_gel_ = 1. Thus, the following expression is used to determine the relative values of the ion mobilities in the gel phase:

(10) $$R_{mem}=\frac{1}{\sum_{i}C_{i}^{gel}\varphi_{i}^{gel}}\;+\frac{Rf_{sol}}{\sum_{i}C_{i}^{sol}\mu_{i}^{sol}}\;\mathrm{with}\;\varphi_{i}^{gel}=\frac{\mu_{i}^{gel}}{Rf_{gel}}$$

The φ_i_^gel^ values and Rf_sol_ are found by fitting the calculated R_gel_ values to the experimental resistances. From the membrane mobilities and membrane concentration, it is now possible to determine the transport numbers in the gel phase for ED or RED applications:

(11) $$t_{i}=\frac{C_{i}^{gel}\varphi_{i}^{gel}}{\sum_{i}C_{i}^{gel}\varphi_{i}^{gel}}$$

Some restrictions of the former procedure are as follows: (i) the concentrations of the co-ions in the gel phase are very low due to the Donnan exclusion, and therefore, the influence of co-ions on the total conductivity of the membrane is marginal and the values obtained for the mobilities are of little validity, and (ii) the same applies to the transport numbers, as calculated with these mobilities. However, the fitting process provides a good insight of the relationship of the mobilities of the counter-ions in the gel phase.

## 4. Results

This section presents and discusses the main results obtained in the application of the ion transport model to describe the influence of divalent ions on membrane resistance.

### 4.1. Estimation of Membrane Resistance

Different membrane resistance measurements were developed according to Table 2 using the EIS method described previously. Equal concentrations on both sides of the membrane were applied. The values with only NaCl salt in the HCS were 5.09 Ω·cm^2^ and 3.20 Ω·cm^2^ for FKS and FAS, respectively, and therefore, cations have more influence in the overall membrane resistance. In this regard, when MgCl_2_ or CaCl_2_ were included in the HCS, the values increased to 10.58 Ω·cm^2^ and 7.97 Ω·cm^2^, respectively. The main reason resides in the fact that when a cation exchange membrane is exposed to a solution containing divalent cations such as Mg^2+^ or Ca^2+^, these cations can bind to the negatively charged functional groups on the membrane, reducing the number of available exchange sites for monovalent cations such as Na^+^. This reduces the number of ions that can pass through the membrane and, therefore, increases the membrane resistance. The reason for this effect is that divalent cations have a higher charge density than monovalent cations, which means they can attract and bind to a larger number of negatively charged functional groups on the membrane. This reduces the number of available exchange sites for monovalent cations, leading to a decrease in the exchange rate and an increase in the membrane resistance. However, including Na_2_SO_4_ does not reflect remarkable changes in the FAS membrane due to the fact that the charge density of SO_4_^2−^ is similar to that of Cl^−^, and both anions can be exchanged by the positively charged functional groups on the membrane.

In the case of including high concentrations of divalent ions in the HCS, in terms of primary divalent ions, the final values were 11.69 Ω·cm^2^ and 3.25 Ω·cm^2^ for FKS and FAS, respectively. In the case of LCS, the values measured were 6.33 Ω·cm^2^ and 3.76 Ω·cm^2^. The upper values of LCS were in accordance with previous values reported in the literature, where membrane resistance increased with the decreasing NaCl concentration [3,4,5,6,7,17,22,41]. Besides, in the case of LCS, when divalent cations were added, 0.0024 M of MgCl_2_ and 0.002 M of CaCl_2_, the membrane resistance sharply increased until 29.39 Ω·cm^2^. In the case of FAS, the membrane resistance grew slightly when 0.0014 M of sodium sulfate was introduced in 0.0186 M of NaCl, but as in the case of HCS, the variation could be considered negligible. The values presented in this work were higher than previous values reported in the literature [22], especially for the cationic exchange membrane, probably due to the reinforcement applied to obtain better stability. However, according to the supplier information, PET promotes more stability at longer periods (years) than other membranes and facilitates the stacking of more membrane pairs. This point is crucial to the system scale-up and to achieve a higher technology readiness level. Moreover, it is important to highlight that the membrane resistance, to extrapolate to salinity gradient extraction, should be calculated as the mean value of high- and low-concentration solutions for each type of membrane, cationic exchange membrane, and anionic exchange membrane, respectively.

### 4.2. Application of the Ion Transport Model

Publications about membrane resistance in solutions containing mixtures of mono- and di-valent ions are very scarce, but the model developed throughout this work has been validated with previously reported data. In Table 3, the main results of the fitting procedure are listed. Normalized mobilities are relative mobilities and are calculated as fractions of all mobilities together. It should be emphasized that the values of the normalized mobilities of the co-ions in the membrane are unreliable, due to their low concentrations, as explained before. The fitting procedure is performed with a number of normalized mobilities and a phase factor; in the case of experiments c and d in Table 3, the total adjustable parameters (k) are 6. Also shown are the coefficients of determination (R^2^). With a restricted number of experiments, the adjusted coefficient of determination (R^2^_adj_) is a better indicator of the performance of the method. It is calculated according to the next equation [42]:

(12) $$R_{\mathrm{adj}}^{2}=1-\frac{\left(1-R^{2}\right)\left(n-1\right)}{n-k-1}$$

Here, n is the number of data points and k is the number of adjustable parameters. An in-depth discussion regarding the main findings is presented below.

Table 3 presents the normalized mobilities of various ions in the gel phase of the membranes under consideration. The use of normalized values allows for better comparison with data from other publications using the mobility ratios of mono- and di-valent counter-ions. Co-ions were not considered due to their low reliability in the fitting procedure as a result of their low concentration in the gel phase. The diffusion constant ratios of the gel phase were obtained using Equation (7) and are presented in Table 3.

Since the ionic mobilities in aqueous solution are comparable (Table 1), the mono/di-valent ratio of diffusion constants in water is approximately 2. For cations, the ratios in the gel phase are expected to be higher due to the larger radius of divalent ions, which restricts their movement. With anions, the difference in the ionic radius is smaller, and the divalent co-ions are more strongly repelled by the fixed charges, leading to less hindered movement. In some cases, this effect can even result in a higher mobility of divalent co-ions than monovalent co-ions. Table 3 also shows values of the diffusion constant ratios.

Although no direct comparison material was found in the literature, Sarapulova et al.’s excellent work [42] provides ĐNa+/ĐCa2+ values of 17, 6, and 2 for the homogeneous membranes CMX, CJMC-3, and CJMC-5, respectively. The Neosepta CXM (Astom Corporation, Japan) can be considered a standard CEM, while both CJMC membranes are relatively new membranes from Hefei Chemjoy Polymer Material Co. Haidian in China.

Data from another paper by Sarapulova et al. [6] were used to obtain the gel diffusion ratios of anions. The values of ĐCl^−^/ĐSO_4_^2−^ for the membranes AMX, AMH-PES, CJMA-3, CJMA-6, and CJMA-7 are 3.3, 2.5, 2.5, 1.4, and 1.7, respectively. The well-known Neosepta AXM (Astom Corporation, Shunan, Japan) and the CJMA membranes (Hefei Chemjoy Pololymer Material Co., Hefei, China) are homogeneous AEMs, while the AMH-PES (Mega a.s., Stráž pod Ralskem, Czech Republic) is a heterogeneous AEM. The diffusion ratios of our studied membranes, FAS-PET-75 and FAS-50 (ĐCl^−^/ĐSO_4_^2−^ is 2.6 and 3.3), fell within this range. However, the value of the AMX membrane (ĐCl^−/^ĐSO_4_^2−^ = infinity) seems to be an outlier.

**Table 3:** Results of the fitting procedure. Values of the normalized mobility of counter-ions in the gel phase of the membrane are shown in blue. Values of co-ions (in red) are less reliable. Also listed are the charge density (CD), the number of data points (n), and the number of adjustable parameters. R^2^ is the coefficient of determination and R^2^_adj_ is the adjusted coefficient of determination. N.R. = not reported. Also shown are the ratios of the mobilities of mono- and di-valent ions in the gel phase and the ratios of the concerning gel diffusion constants.

Exp.	Membrane	Type	CD (eq./L)	Ref.	Method	Salts Employed					n	k	R^2^	R^2^adj.	Normalizad Mobility in the Gel Phase					Ratio Counter-Ion Mobility in the Gel Phase			Ratio Diffusion Constants in the Gel Phase		
						NaCl	MgCl_2_	CaCl_2_	Na_2_SO_4_	MgSO_4_					Na^+^	Mg^2+^	Ca^2+^	Cl^−^	SO_4_^2−^	Na^+^/Mg^2+^	Na^+^/Ca^2+^	Cl^−^/SO_4_^2−^	Na^+^/Ca^2+^	Cl^−^/SO_4_^2−^	Na^+^/Ca^2+^
a	CMX	CEM	9	[33]	AC	+	−	−	−	−	7	3	0.999	0.999	0.976	−	−	0.024	-	-	-	−	−	−	−
b	CMX	CEM	9	[33]	DC	+	−	−	−	−	7	3	0.999	0.999	1	−	−	0	-	-	-	−	−	−	−
c	FKS-PET-75	CEM	5.5	This work	EIS	+	+	+	+	−	10	6	0.997	0.99	0.771	0.085	0.116	0.029	0	9.1	6.7	−	18.2	13.4	−
d	FAS-PET-75	AEM	9	This work	EIS	+	+	+	+	−	10	6	0.953	0.859	0.22	0.754	0	0.014	0.011	-	-	1.3	−	−	2.6
e	FKS-50	CEM	8.7	[22]	EIS	+	+	+	−	−	18	5	0.9233	0.891	0.679	0.151	0.17	0	-	4.5	4	-	9	8	−
f	FAS-50	AEM	10.3	[22]	EIS	+	−	−	+	−	10	4	0.964	0.935	0.844	−	−	0.098	0.059	−	−	1.7	−	−	3.3
g	Fuji-CEM-80050	CEM	2.4	[15]	EIS	+	+	−	−	−	7	4	0.922	0.767	0.635	0	−	0.365	-	∞	−	−	∞	−	−
h	CMX	CEM	9	[18]	NR	+	−	−	−	+	6	5	0.998	−	0.315	0.033	−	0	0.652	9.5	−	−	19	−	−
i	AMX	AEM	7.8	[18]	NR	+	−	−	−	+	6	5	0.95	−	0.994	0	−	0.006	0	−	−	∞	−	−	∞

#### 4.2.1. Fumatech Membranes

Two types of membranes with different thicknesses from the same manufacturer, Fumatech, were evaluated following the model procedure. In the first case, the fitting was employed to predict the values obtained experimentally using FKS-PET-75 and FAS-PET-75.

**Figure 3:**
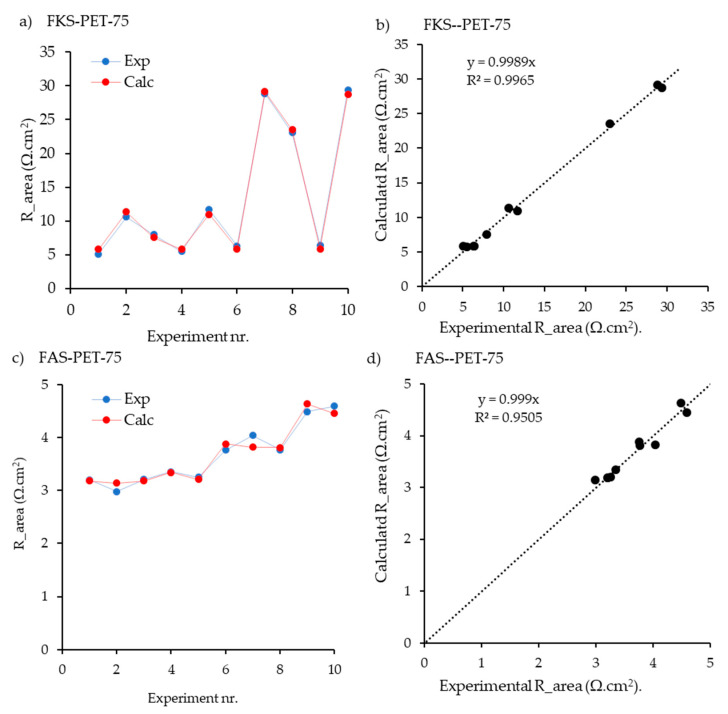
Results of the fitting procedure for the Fumatech membranes FKS-PET-75 (**a**,**b**) and FAS-PET-75 (**c**,**d**), with solutions containing NaCl, MgCl_2_, CaCl_2_, and Na_2_SO_4_. The composition of the various solutions is indicated with “Experiment nr.” and refers to Table 2 (data from this publication). In (**a**,**c**), lines are added to guide the eye; in (**b,d**) the regression lines are shown.

The input data used for the model development are collected in Table 2. Different combinations of NaCl, MgCl_2_, CaCl_2_, and Na_2_SO_4_ were prepared for the estimation. In Figure 3a,b, the experimental and calculated values of R_area_ are plotted for the various experiments, as tabulated in Table 2, whereas in Figure 3b,d, the correlations between the calculated and experimental values are shown. The figures show that the calculated values practically overlap the experimental values. However, despite the fitting being adequate, the relatively limited number of measurements (7) compared to the parameters to be adjusted (5 ion mobilities plus a phase factor) require future work to support model predictions. Besides, it is essential to remark that the parity plot demonstrates the high R^2^ obtained for both AEM and CEM.

On the other hand, membranes with a thickness of 50 µm, FKS-50 and FAS-50 without reinforcement, were evaluated, taking the data from Gómez-Coma et al. [22]. In this previous work, the resistance of FKS-50 and FAS-50 membranes (Fumatech) was determined by EIS measurements between mercury electrodes with a measuring cell equipped with a system to completely remove air bubbles between the membrane and the electrode. The ions evaluated were the same as for the membranes of 75 µm: NaCl, MgCl_2_, CaCl_2_, and Na_2_SO_4_. From the reported data, IEC = 1.2–1.4 meq/g and SD = 10–10% [43], a CD = 8.67 eq/L was achieved from the mean values.

**Figure 4:**
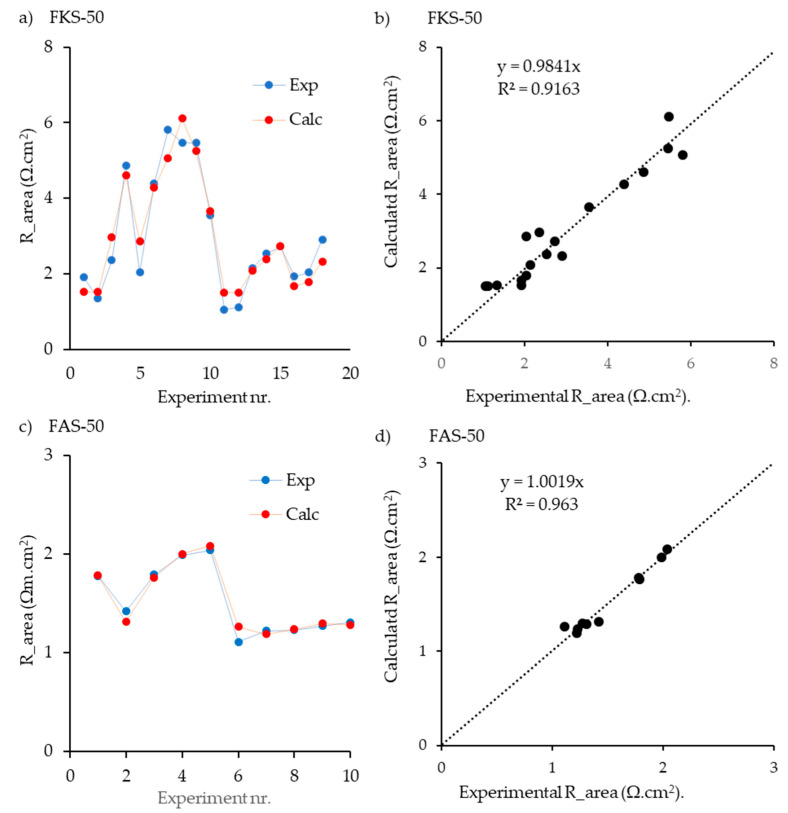
Results of the fitting procedure for the Fumatech membranes FKS-50 (**a**,**b**) and FAS-50 (**c**,**d**). Used were solutions containing NaCl, MgCl_2_, CaCl_2_, NaCl, and Na_2_SO_4_. Data are from Gόmez-Coma et al. [22]. In (**a**,**c**), lines are added to guide the eye; in (**b**,**d**) the regression lines are shown.

As Figure 4 shows, there was a good correlation between the model and the experiment, especially in the case of anionic membranes. In this sense, 10 independent tests were analyzed, obtaining errors lower than 15% in all scenarios. Besides, FKS membranes also demonstrated high accuracy in a series of 19 experiments. Another key point is the difference in membrane resistance when a 75 µm thickness with reinforcement was used. Using 50 µm, all the values were under 6 Ω·cm^2^ in the case of cationic membranes and below 2 Ω·cm^2^ for the anionic membranes. However, in the case of 75 µm, all the values were above 5 Ω·cm^2^ and 3 Ω·cm^2^ for cationic and anionic membranes, respectively.

#### 4.2.2. Results with Neosepta CMX with AC and DC

**Figure 5:**
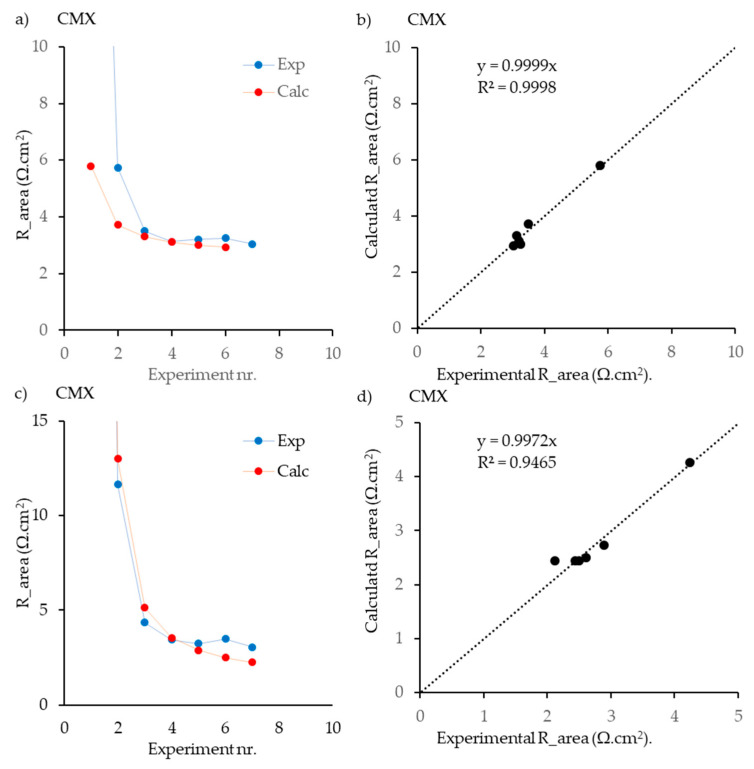
Results of the fitting procedure for the CMX membrane in pure NaCl solutions. (**a**,**b**) Derived from AC measured resistances and (**c**,**d**) from DC measurements from Galama et al. [33]. In (**a**,**c**), lines are added to guide the eye; in (**b,d**) the regression lines are shown.

In this section, the model proposed to calculate the membrane resistance was first compared to the data provided by Galama et al. [33]. The resistance of a Neosepta CMX membrane (Tokuyama, Chiyoda City, Japan) was determined in a six-compartment cell with EIS (Figure 5a,b) and with a direct current (DC) (Figure 5c,d). Experiments were performed with pure NaCl solutions. These data were used to check predicted data with the model reported here. According to Długołęcki et al. [44], the IEC is 1.62 meq/g and SD = 18%, resulting in a CD = 1.62/0.18 = 9.00. Following the procedure in the Section 1, a good fitting with a coefficient of determination of R^2^ = 0.999 for the alternating current (AC) was obtained, taking into consideration the last five values since the first two showed a high difference, probably due to the low concentration of salt employed. However, experiments from 3 to 7 were practically overlapped, as Figure 5 confirms. On the other hand, the DC experiments matched in all the cases with high precision, achieving an R^2^ = 0.997.

#### 4.2.3. Results with Fuji-CEM-80050, Neasepta CMX, and Neosepta AMX

**Figure 6:**
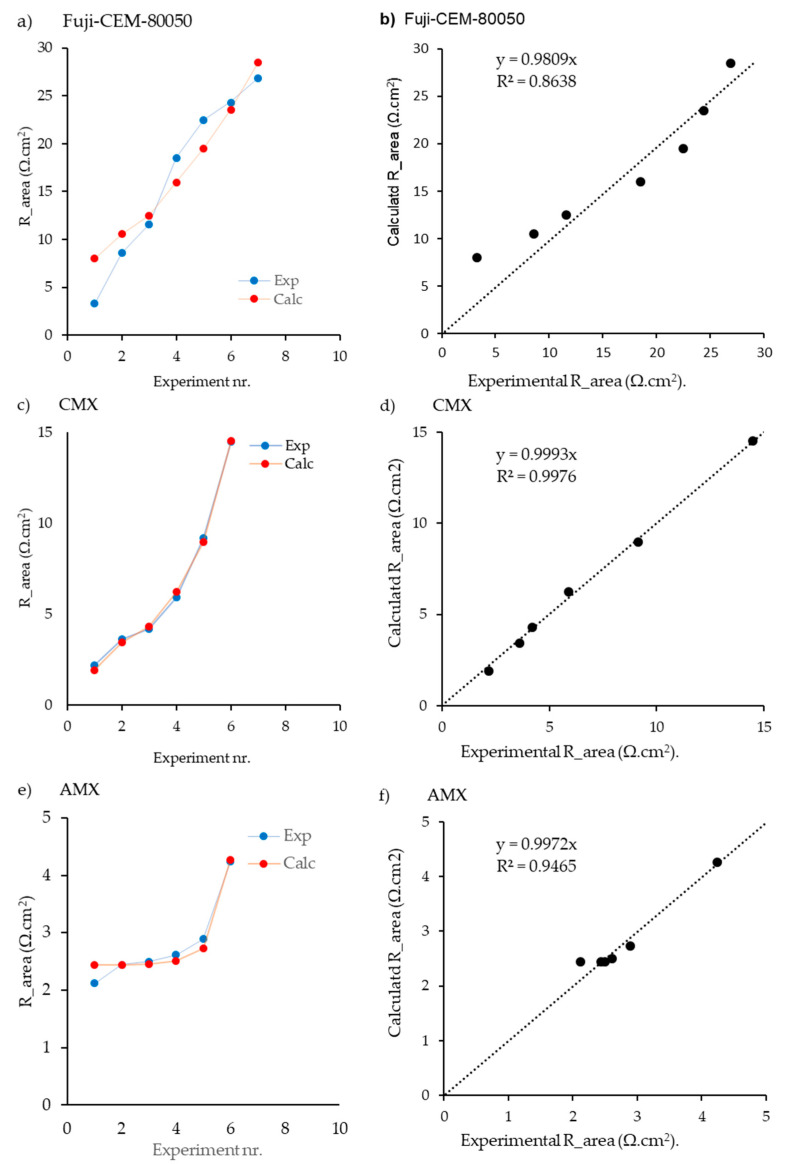
Results of the fitting procedure for Fuji-CEM-80050 (Fujifilm), CMX (Neosepta), and AMX (Neosepta). In (**a**,**c**,**e**), lines are added to guide the eye; in (**b**,**d**,**f**) the regression lines are shown.

In contrast, the data for the Fuji-CEM-80050, provided by Fujifilm, were obtained from Avci et al. [15] using the total resistance (the resistance of the membrane, diffusion boundary layer, and electric double layer, together). The resistances were determined using EIS in a four-electrode cell configuration. In this case, the results exhibited a reasonably good agreement in the comparison of 7 different concentrations. On the other hand, and as it can be confirmed through Figure 6b, in the case of cationic membranes of Fuji-CEM-80050, the model fit better to higher values of membrane resistance, which implies lower values of NaCl and higher values of MgCl_2_ (Table S4).

Finally, CMX and AMX membranes from Neosepta were studied, in this case working with data reported by Kuno et al. [18], which demonstrated the best level of prediction, between the experimental and simulated values fitting with an R^2^ value higher than 0.99 for both types of membranes (Figure 6). As in the previous cases, cation exchange membranes showed higher resistance values than anionic membranes, probably due to the high selectivity of AMX [42].

### 4.3. Gross Power Density

**Figure 7:**
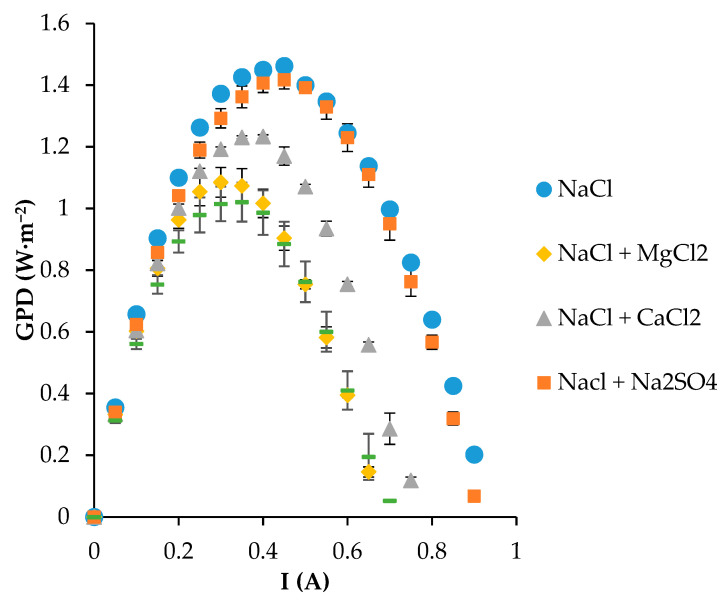
Gross power density achieved with NaCl and divalent ions.

To better understand the impact of the membrane resistance, different trials in a RED module for salinity gradient energy extraction (which is highly dependent on IEMs’ contribution) have been performed. Regarding the gross power obtained per membrane pair, Figure 7 depicts the results of working with 20 membrane pairs of FKS-75 and FAS-75. As observed, when only sodium chloride was included in the feed streams in the typical composition of seawater (0.5 M) and wastewater treatment plants (0.02 M), the gross power density (GPD) achieved values as high as 1.46 W·m^−2^. This value concurs with previous works reported in the recent literature for similar membranes based on the same polymer but with different membrane thicknesses [38]. This is because of a positive impact in the membrane’s thickness but a negative impact in the increment of the membrane resistance [32,36,43]. Moreover, the presence of Na_2_SO_4_ in the sample did not reflect changes in the GPD since the value was practically the same (1.42 W·m^−2^) as when only NaCl flowed through the compartments. This point is probably related to the fact that the conductivity of a Na_2_SO_4_ solution drops sharply with the increasing salt concentration [44]. On the other hand, the inclusion of MgCl_2_ impacted the salinity power density, which decreased to 1.08 W·m^−2^. The reason for the increase in resistance was previously attributed to the lower mobility (due to a higher hydrated radius) of Mg^2+^ than Na^+^, which contributed to the loss of permselectivity [45]. In addition, the effect of CaCl_2_ was very high since with the inclusion of 0.011 and 0.002 M in the high and low concentrations, respectively, the total gross power decay was 16%. This point matches with previous results reported in the literature, where the effective order of coexisting ions on the energy generation performance was Ca^2+^ > Mg^2+^ > SO_4_^2−^ [46]. On the other hand, it is important to remark that Mg^2+^ and Ca^2+^ displayed a negligible effect on the resistance of AEM due to the Donnan exclusion [45,47]. However, as expected, and according to the membrane resistance, when all the typical divalent ions were included, the gross power density was 1.02 W·m^−2^. Despite the strong decay with respect to the use of NaCl, these values were higher or in the same range as previous works reported in the literature [48,49,50].

In all the cases, after the experiments, the RED module was opened to check the membranes and spacers, ensuring no fouling marks. Moreover, the NaCl test was replicated after each divalent trial to confirm the stability of the GPD.

## 5. Conclusions

This work reported an in-depth analysis of the impact of divalent ions on ion exchange membranes, in terms of membrane resistance, to better understand the influence in reverse electrodialysis technology. In this sense, the results demonstrated the high impact on the resistance values of the presence of cations, and the quite negligible influence of anions. While the anionic exchange membrane exhibited values of 3.25 and 4.59 Ω·cm^2^ for HCS and LCS, respectively, the cation exchange membrane displayed values of 11.69 and 29.39 Ω·cm^2^. In a second step, a model to predict the membrane resistance has been developed, considering the transport phenomena of ions in complex mixtures, achieving high correlations between simulation and experimental values. However, this work also demonstrated the difficulty to standardize the determination of the membrane resistance and membrane potential for different water compositions. Furthermore, this work showed that even in the case of pure NaCl, there were large differences in the measured resistance [4] due to major variations in pre-treatment methods, in the equipment employed, and in the method chosen for the measures. Thus, despite the strong contribution of membrane resistance in different sectors, such as renewable energies, for example, salinity gradient energy extraction, the development of a universal method for all the market membranes is far from being possible. Additionally, this work presented an evaluation of the extraction of the salinity gradient power using RED technology, using 20 membrane cell pairs and woven spacers at different water compositions, and therefore different membrane resistances. Specifically, the value reported for power density was 1.02 W·m^−2^, when a certain not insignificant number of divalent ions were added, which was one of the highest values found for waters in the presence of divalent ions in the recent literature. The results presented and discussed here constitute a step forward for the scale-up of the process and the understanding of the contribution of ion exchange membrane resistance to the overall process performance.

## Figures and Tables

**Table 1:** Equivalent mobilities in water at 298.15 K [39].

Cation	λ (m^2^∙s^−1^∙V^−1^)	Anion	λ (m^2^∙s^−1^∙V^−1^)
Na^+^	5.20 × 10^−8^	Cl^−^	7.90 × 10^−8^
Mg^2+^	5.50 × 10^−8^	SO_4_^2−^	8.27 × 10^−8^
Ca^2+^	6.16 × 10^−8^		

**Table 2:** Membrane resistance values (Ω·cm^2^) for FKS and FAS in high- and low-concentration solutions (M).

	Experiment	M				Membrane Resistance (Ω·cm^2^)	
	No.	NaCl	MgCl_2_	CaCl_2_	Na_2_SO_4_	FKS-PET-75	FAS-PET-75
HCS	1	0.5	0	0	0	5.09	3.20
	2	0.49	0.06	0	0	10.58	2.98
	3	0.49	0	0.011	0	7.97	3.21
	4	0.49	0	0	0.032	5.49	3.35
	5	0.49	0.06	0.011	0.032	11.69	3.25
LCS	6	0.02	0	0	0	6.33	3.76
	7	0.0176	0.0024	0	0	28.86	4.04
	8	0.0185	0	0.002	0	23.05	3.77
	9	0.0186	0	0	0.0014	6.40	4.49
	10	0.0172	0.0024	0.002	0.0014	29.39	4.59

## Supplementary Materials

The following supporting information can be downloaded at: https://www.mdpi.com/article/10.3390/membranes13030322/s1. Table S1. Resistances of FKS-50 measured at 297 ± 1 K. Table S2. Resistances of FAS-50 measured at 297 ± 1 K. Table S3. Resistances of CMX measured at 298 K with direct current (DC) and with alternating current (AC). Table S4. Resistances of Fuji-CEM-80050 CMX measured at 298 K. Table S5. Resistances of AMX and CMX [5,15,18,22,33,44,51].

## Nomenclature

Roman	
A	Membrane areas (cm^2^)
C	Concentration (eq/L)
CD	Membrane charge density (eq/L)
D	Diffusion constant in H_2_O (m^2^/s)
Đ	Diffusion constant in gel phase (m^2^/s)
d	Membrane thickness (cm)
F	Faraday constant (96,485 C∙mol^−1^)
IEC	Ion exchange capacity (meq/g dry membrane)
SD	Membrane swelling degree (−)
k	Boltzmann constant (1.381 × 10^−23^ J/K)
K	Donnan equilibrium constant (−)
R_area_	Membrane area resistance (Ω∙m^2^)
Rfgel	Resistance factor of the gel part
Rfsol	Resistance factor of the solution part
R_gel_	Resistance of the gel part (Ω)
R_mem_	Resistance of the membrane (Ω)
R_sol_	Resistance of the solution part (Ω)
R_spec_	Specific resistance (Ω∙m)
Q	Ion charge (C)
T	Temperature (K)
z	Ionic valence
Greek	
φ	Relative ionic mobility (−)
κ	Conductivity (S/m)
λ	Ionic mobility (m^2^∙s^−1^∙V^−1^)
ρ	Density of the dry membrane (kg/L)
Sub- and super-scripts	
gel	Gel phase
sol	Solution phase
i	Ionic species i
co	Co-ions
ct	Counter-ions
Acronyms	
AEM	Anion exchange membrane
AC	Alternating current
CEM	Cation exchange membrane
DC	Direct current
EIS	Electrochemical impedance spectrometry
GPD	Gross power density
HCS	High-concentration solution
IEM	Ion exchange membranes
LCS	Low-concentration solution
RED	Reverse electrodialysis
