# Supplementary material for: Resistance of Ion Exchange Membranes in Aqueous Mixtures of Monovalent and Divalent Ions and the Effect on Reverse Electrodialysis
Source: Membranes (Basel). 2023 Mar 10;13(3):322. doi: 10.3390/membranes13030322 (PMC10056131; doi:10.3390/membranes13030322)
Supplement: Supplementary file 1 [file membranes-13-00322-s001.zip › membranes-2195279-supplementary.pdf]

membranes

MDPI

Supporting Information to: Resistance of ion exchange membranes in aqueous mixtures of monovalent and divalent ions and the effect on reverse electrodialysis

Joost Veerman [1], Lucía Gómez-Coma [2], Alfredo Ortiz [2] and Inmaculada Ortiz [2,*]

[1] REDstack bv, Graaf Adolfstraat 35-G, 8606 BT Sneek, The Netherlands
[2] Department of Chemical and Biomolecular Engineering. University of Cantabria, Av. Los Castros 46, 39005 Santander, Spain
* Correspondence: ortizi@unican.es

Citation: Veerman, J.; Gómez-Coma, L.; Ortiz, A.; Ortiz, I. Resistance of Ion Exchange Membranes in Aqueous Mixtures of Monovalent and Divalent Ions and the Effect on Reverse Electrodialysis. *Membranes* **2023**, *13*, 322. https://doi.org/10.3390/membranes13030322

Academic Editors: Liliana C. Tomé, Luisa A. Neves and Isabel Coelhoso

Received: 16 January 2023
Revised: 1 March 2023
Accepted: 6 March 2023
Published: 10 March 2023

Copyright: © 2023 by the authors. Licensee MDPI, Basel, Switzerland. This article is an open access article distributed under the terms and conditions of the Creative Commons Attribution (CC BY) license (https://creativecommons.org/licenses/by/4.0/).

1.Composition of the solutions used for testing FKS-50 and FAS-50 as presented in Figure 5.
Source: Gómez-Coma et al. 2019 [1].

Exp	NaCl (M)	$MgCl_2$ (M)	$CaCl_2$ (M)	Rarea (Ω·cm²)
1	$6\cdot10^{-4}$	0	0	1.92
2	0.02	0	0	1.34
3	0.02	$1\cdot10^{-4}$	0	2.36
4	0.02	$1\cdot10^{-3}$	0	4.87
5	0.02	0	$1\cdot10^{-4}$	2.04
6	0.02	0	$1\cdot10^{-3}$	4.40
7	0.02	0	$5\cdot10^{-3}$	5.81
8	$6\cdot10^{-4}$	$2.86\cdot10^{-4}$	$1.19\cdot10^{-3}$	5.47
9	0.02	$2.4\cdot10^{-3}$	$1.5\cdot10^{-3}$	5.46
10	0.043	$1.1\cdot10^{-3}$	$2.0\cdot10^{-4}$	3.56
11	0.55	0	0	1.05
12	1	0	0	1.11
13	1	0.05	0	2.14
14	1	0.1	0	2.53
15	1	0.2	0	2.72
16	1	0	0.01	1.93
17	1	0	0.02	2.03
18	1	0	0.1	2.91

Table S1. Resistances of FKS-50 measured at 297±1 K

Exp	NaCl (M)	Na_2SO_4 (M)	R_{area} (Ω·cm^2)
1	$6 \cdot 10^{-4}$	0	1.78
2	0.02	0	1.42
3	0.02	$1 \cdot 10^{-4}$	1.79
4	0.02	$1 \cdot 10^{-3}$	1.99
5	0.02	$5 \cdot 10^{-3}$	2.04
6	0.55	0	1.11
7	1	0	1.22
8	1	0.01	1.23
9	1	0.06	1.27
10	1	0.1	1.31

Table S2. Resistances of FAS-50 measured at 297±1 K

2. Composition of the solutions used for testing CMX as presented in Figure 6.

Source**:** Galama et al. 2014 [2].

Exp	NaCl (M)	R_{area} (Ω·cm^2) DC	R_{area} (Ω·cm^2) AC
1	0.01	119.47	33.78
2	0.1	11.65	5.74
3	0.3	4.35	3.50
4	0.5	3.43	3.13
5	0.7	3.24	3.21
6	0.9	3.47	3.25
7	1.1	3.41	3.04

Table S3. Resistances of CMX measured at 298 K with direct current (DC) and with alternating current (AC).

3. Composition of the solutions used for testing Fuji-CEM-80050 as presented in Figure 7.

Source: Avci et al. 2016 [3].

Exp	NaCl (M)	$MgCl_2$ (M)	R_{area} (Ω·cm^2)
1	1	0	3.284
2	0.9	0.1	8.579
3	0.8	0.2	11.6
4	0.6	0.4	18.5
5	0.4	0.6	22.52
6	0.2	0.8	24.4
7	0	1	26.88

Table S4. Resistances of Fuji-CEM-80050 CMX measured at 298 K.

4. Composition of the solutions used for testing AMX and CMX as presented in Figure 7.

Source**:** Kuno et al. 2017 [4].

Exp	NaCl (M)	$MgSO_4$ (M)	R_{area} (Ω·cm^2) AMX	R_{area} (Ω·cm^2) CMX
1	0.508	0	2.122	2.175
2	0.4826	0.0254	2.443	3.61
3	0.4572	0.0508	2.499	4.196
4	0.381	0.127	2.613	5.902
5	0.254	0.254	2.891	9.151
6	0	0.508	4.244	14.48

Table S5. Resistances of AMX and CMX. .

5. Specifications of the discussed membranes.

Specs	FAS-50	FKS-50	FAS-PET-75	FKS-PET-75	CMX	AMX	Fuji-CEM-80050
Manu-facturer	Fumatec BWT GmbH, Germany				ASTOM Co., Ltd, Japan		Fujifilm B.V. The Netherlands
Type	AEM	CEM	AEM	CEM	AEM	CEM	CEM
Ref.	[5]	[5]	[5]	[5]	[6] [7]	[6] [7]	[4]
Exchange group	~Br^-	~H+	~Br^-	~H+	~SO_3^-	~$N^+(CH_3)_3$	~$N^+(CH_3)_3$
Backbone	PET	PET	PET	PET	Styrene + divinylbenzene		polyolifin
Reinforce-ment	no	no	PET	PET	PVC		Electrospun nanofibers
Thickness (μm)	45 - 55	45 - 55	70-80	74 - 87	164	134	114 ± 2
IEC (meq/g)	1.6 - 2.0	1.2 - 1.4	1.2-1.4	1.0 - 1.25	1.62	1.25	1.1 ± 0.1
R_{area} (Ωcm^2)	0.6 - 1.5	1.8 - 2.5	1.2-2.0	< 2.5	2.91	2.35	3.0
Perm-selectivity (%)	92 - 96	97 - 99	94-97	> 95	99.0	90.7	-
H_2O up-take (%)	10 - 25	10 - 20	15-25	-	18	16	-
CD meq/gH_2O	14.4	8.o	12.0	-	9.0	7. 8	2.4

6. Experimental techniques

Applied	Gómez-Coma et al.	Gómez-Coma et al.	Galama et al.	Kuno et al.	Avci et al.
Ref.	[1]	This paper	[2]	[4]	[3]
Membranes	FAS-50<br>FKS-50	FAS-PET-75<br>FKS-PET-75	CEM	AMX<br>CMX	Fuji-CEM-80050
Preconditioning	.	24 h	3 x 24 h	?	3 x 8 h
Temp. (K)	297	297	298	?	298
Compartments .in cell	2	2	6	2	2
Work /counter electrodes	Hg<br>Direct contact	Hg<br>Direct contact	.Pt covered Ti	?	Ag/AgCl covered Ag
Reference elec-trodes	no	no	Ag/AgCl via Haber-Luggin capillaries	no	Ag/AgCl via Haber-Luggin capillaries

References

[1] Gómez-Coma, L.; Ortiz-Martínez, V.M.; Carmona, J.; Palacio, L.; Prádanos, P.; Fallanza, M.; Ortiz, A.; Ibañez R.; and Ortiz, I. "Modeling the influence of divalent ions on membrane resistance and electric power in reverse electrodialysis," *Journal of Membrane Science*, 2019, 592, 117385, https://doi.org/10.1016/j.memsci.2019.117385.

[2] Galama, A.H.; Vermaas, D.A.; Veerman, J.; Saakes, M.; Rijnaarts, H.H.M.; Post J.W.; and Nijmeijer, K. "Membrane resistance: The effect of salinity gradients over a cation exchange membrane," *Journal of Membrane Science*, 2014, 467, 279-291; https://doi.org/10.1016/j.memsci.2014.05.046.

[3] Avci, A.H.; Sarkar, P.; Tufa, R.A.; Messana, D.; Argurio, P.; Fontananova, E.; Di Profio G.; and Curcio, E. "Effect of Mg^{2+} ions on energy generation by reverse electrodialysis," *Journal of Membrane Science* 2016, 520, 499-506; http://dx.doi.org/10.1016/j.mem-sci.2016.08.007.

[4] Kuno, M.; Yasukawa, M.; Kakihana Y.; and Higa, M. " The effect of divalent ions on reverse electrodialysis power generation system," *Bull. Soc. Sea Water Sci., Jpn.,* 2017, 71, 350- 351.

[5] https://www.fuelcellstore.com/fuel-cell-components/membranes (accessed on 24 January 2023).

[6] Długołęcki, P.; Nymeijer, K.; Metz, S.; and Wessling, M. "Current status of ion exchange membranes for power generation from salinity gradients," *Journal of Membrane Science 319-1,2 (2008) 214-222;* https://doi.org/10.1016/j.memsci.2008.03.037.

[7] V. Sarapulova, I. Shkorkina, S. Mareev, N. Pismenskaya, N. Kononenko, C. Larchet, L. Dammak and V. Nikonenko, "Transport Characteristics of Fujifilm Ion-Exchange Membranes as Compared to Homogeneous Membranes AMX and CMX and to Heterogeneous Membranes MK-40 and MA-41," *Membranes 9-7 (2019) 84; doi:10.3390/membranes9070084.*

Disclaimer/Publisher's Note: The statements, opinions and data contained in all publications are solely those of the individual author(s) and contributor(s) and not of MDPI and/or the editor(s). MDPI and/or the editor(s) disclaim responsibility for any injury to people or property resulting from any ideas, methods, instructions or products referred to in the content.
